# Multi-modal and multiscale imaging approaches reveal novel cardiovascular pathophysiology in *Drosophila melanogaster*

**DOI:** 10.1242/bio.044339

**Published:** 2019-08-15

**Authors:** Constance G. Weismann, Anna Blice-Baum, Tangji Tong, Joyce Li, Brendan K. Huang, Stephan M. Jonas, Anthony Cammarato, Michael A. Choma

**Affiliations:** 1 Yale School of Medicine, Department of Pediatrics, Division of Pediatric Cardiology, New Haven, CT 06510, USA; 2 Lund University, Skane University Hospital, Department of Clinical Sciences Lund, Pediatric Cardiology, 22184 Lund, Sweden; 3 Johns Hopkins University School of Medicine, Division of Cardiology, Department of Medicine, Department of Physiology, Baltimore, MD 21205, USA; 4 Yale Departments of Diagnostic Radiology, Pediatrics, Biomedical Engineering, and Applied Physics, New Haven, CT 06510, USA; 5 Department of Informatics, Technical University of Munich, 85748 Garching, Germany

**Keywords:** *Drosophila melanogaster*, OCT, Aortic stiffness, Cardiovascular physiology, *hdp^2^*, Troponin

## Abstract

Establishing connections between changes in linear DNA sequences and complex downstream mesoscopic pathology remains a major challenge in biology. Herein, we report a novel, multi-modal and multiscale imaging approach for comprehensive assessment of cardiovascular physiology in *Drosophila melanogaster*. We employed high-speed angiography, optical coherence tomography (OCT) and confocal microscopy to reveal functional and structural abnormalities in the *hdp^2^* mutant, pre-pupal heart tube and aorta relative to controls. *hdp^2^* harbor a mutation in *wupA*, which encodes an ortholog of human troponin I (*TNNI3*). *TNNI3* variants frequently engender cardiomyopathy. We demonstrate that the *hdp^2^* aortic and cardiac muscle walls are disrupted and that shorter sarcomeres are associated with smaller, stiffer aortas, which consequently result in increased flow and pulse wave velocities. The mutant hearts also displayed diastolic and latent systolic dysfunction. We conclude that *hdp^2^* pre-pupal hearts are exposed to increased afterload due to aortic hypoplasia. This may in turn contribute to diastolic and subtle systolic dysfunction via vascular-heart tube interaction, which describes the effect of the arterial loading system on cardiac function. Ultimately, the cardiovascular pathophysiology caused by a point mutation in a sarcomeric protein demonstrates that complex and dynamic micro- and mesoscopic phenotypes can be mechanistically explained in a gene sequence- and molecular-specific manner.

## Abstract (precis)

**Summary:** Mutant troponin I increases aortic and myocardial stiffness and engenders histologic abnormalities in fruit flies.

## INTRODUCTION

Establishing connections between changes in linear DNA sequences and downstream mesoscopic pathology remains a major challenge in biology, especially when the ensuing defects involve complex physiological motions and fluid flows. For example, in cardiovascular physiology, mutations in sarcomeric genes impact multiple levels of the cardiovascular system, from nanoscopic molecular function to myofiber organization and ultimately dynamic pump performance of the heart. While the sophistication of genetic manipulation of animal models of cardiovascular disease continues to progress, multiscale characterization of resultant pathophysiology remains largely lacking. Multiscale physiology seeks to integrate molecular genetic information with nanoscopic and constitutive properties at the tissue and organ level. Different optical imaging modalities can yield insight across diverse size and time scales. There are a growing number of such imaging methods available that exploit a number of endogenous and exogenous contrast mechanisms, which lend themselves to the quantification of many physiological parameters. Several optical approaches also exploit high-speed frame rates for well-resolved *in vivo* imaging. Nevertheless, the use of multimodal optical imaging to yield insight into multiscale physiology remains in its infancy. Moreover, the downstream functional consequences of DNA defects in sarcomeric genes are difficult to predict because (1) baseline micro- and mesoscopic physiology is not completely known and (2) there are no adequate multiscale models to assess such predictions. Thus, establishing direct multiscale imaging is indispensable in studying specific biomedically relevant questions of cardiovascular physiology.

The use of cardiovascular systems of organisms with body lengths in the millimeter regime presents an intriguing opportunity for multiscale, multimodal optical imaging. Such animals permit cardiovascular pathophysiology assessment in a quantitative, hierarchical manner ranging from the functional unit to whole-organism physiology. The hierarchy scales from nano/microscopic sarcomere function to microscopic heart and aortic wall motion and mesoscopic fluid flow. This range of physiology is incompletely understood.

*Drosophila melanogaster*, the fruit fly, is an attractive organism for studying cardiovascular performance*.* In flies as well as in vertebrate embryos during early development, a cardiac chamber pumps into a muscular vessel (*D. melanogaster*, aorta; vertebrate embryo, outflow tract). The conduits are distinct from arterial blood vessels and from an ontological, functional and ultrastructural perspective, are more closely related to cardiac chambers. The muscular vessels are composed of striated rather than smooth muscle as present in vertebrate systemic vasculature. Flies are an established animal model of muscle physiology and cardiovascular disease. Multiple sarcomere-related mutant strains exist that offer distinct opportunities to study the micro- to mesoscopic physiology of hearts pumping into muscular outflow vessels. Prior work in *D. melanogaster* has established several imaging approaches for multiscale, multimodal imaging (Choma et al., 2011; Ocorr et al., 2007; Viswanathan et al., 2014; Wolf et al., 2006), although such work was often limited in its ability to connect abnormalities in fast micro- and mesoscopic physiology to underlying defects in sarcomeres. Here, we integrate data extracted from multimodal optical imaging into a novel, multiscale view of cardiovascular functional defects in the setting of a sarcomeric gene mutation in *D. melanogaster*. Specifically, we studied the coupled heart tube-aorta physiology in *held-up^2^* (*hdp^2^*) pre-pupae. *hdp^2^* flies harbor a mutation in the *wings up A* (*wupA*) gene that results in an A55V substitution in the troponin I (TNNI3, OMIM #191044) ortholog (Beall and Fyrberg, 1991). Mutations in *TNNI3* typically cause hypertrophic cardiomyopathy in humans, although some lesions have also been associated with restrictive and dilated cardiomyopathies (Mogensen et al., 2015). A cardiomyopathy phenotype in adult flies and abnormal physiology in pre-pupal *hdp^2^* hearts have previously been shown (Choma et al., 2011; Wolf et al., 2006). Micro- and mesoscopic imaging, using structural and Doppler optical coherence tomography (OCT) and high-speed (250–500 frames per second) optical angiography revealed that *hdp^2^* pathophysiology is characterized by a hypoplastic aortic phenotype with associated dynamic structural as well as fluid flow defects. To complement structural and functional heart and aortic imaging, we used confocal microscopy to image and quantify sarcomeric structure and dimensions, which revealed that *hdp^2^* pre-pupal hearts and aortas have fewer longitudinal myofibrils and shorter sarcomeres. This is consistent with (a) prior observations that *hdp^2^* muscle is hypercontractile and in a disinhibited state (Beall and Fyrberg, 1991; Cammarato et al., 2004) and (b) our new observation that hypoplastic aortas have reduced diameter and increased fluid flow velocities. Overall, our approach and results argue for expanded use of multiscale, multimodal optical imaging in cardiovascular physiology.

## RESULTS

### High-speed optical angiographic imaging of baseline mesoscopic cardiovascular physiology in pre-pupal *D. melanogaster*

**Fig. 1.**
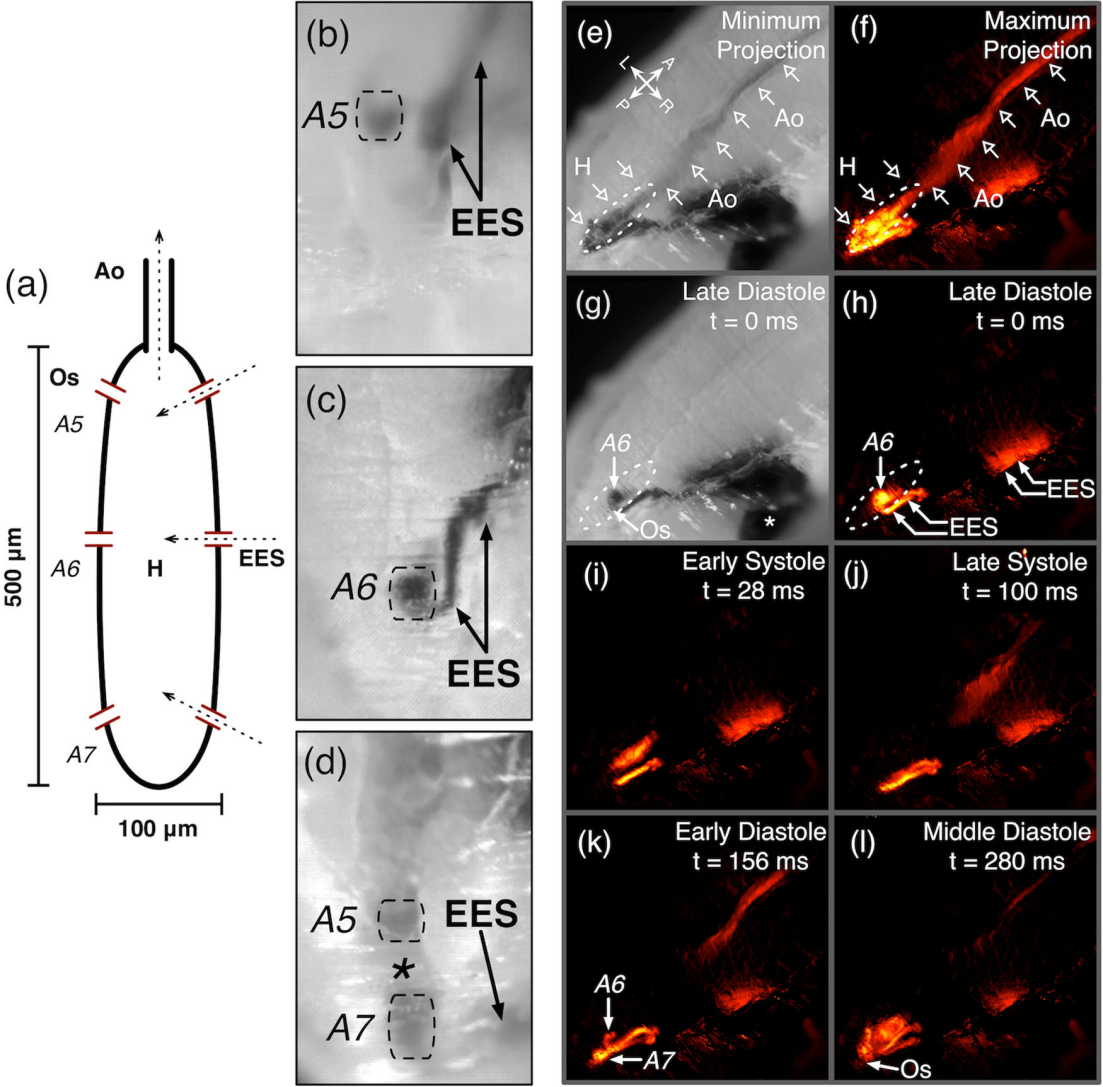
**High-speed optical angiography of pre-pupal *D. melanogaster* cardiac and aortic fluid flow.** (A) Schematic of the pre-pupal *D. melanogaster* heart (H) and proximal aorta (Ao). The heart has three pairs of intake valves (ostia, Os). Each pair can be identified by its abdominal segmental anatomic location: A5, A6 or A7. Scale bars depict the typical cardiac size of 500×100 µm. (B–D) Unilateral dye inflow though A5 (B, Movie 1), A6 (C, Movie 2) and A7 (D, Movie 3) ostia. Dye injected into the extracellular-extravascular space (EES) is transported into the heart during diastole. In (D) and Movie 3, dye entered through an A7 ostium and residual dye in the heart after one cardiac cycle remained in the A5 segment of the heart. The hemolymph in the A6 region (*) remained unenhanced by dye. (E–F) Visualization of cardiac and aortic flow over one cardiac cycle using optical angiography and background-free digital subtraction angiography (second cardiac cycle in Movie 4). Projection images E and F highlight the luminal enhancement over the cardiac cycle. (G–L) show representative stages of the cardiac cycle that are captured with 4 ms temporal resolution. A, anterior; P, posterior; L, left; R, right; * in G, dye injection site.

High-speed optical angiography was used to gain insight into mesoscopic flow physiology in wild-type *Oregon Red* (*OreR*) pre-pupal *D. melanogaster* (Fig. 1, Movies 1–5). We injected a dye solution (Brilliant Blue G) into the extracellular-extravascular space using custom-pulled glass pipettes (Choma et al., 2011). We imaged at 250–500 frames per second, much faster than our prior angiographic work in the video rate regime. To enable background-free visualization of angiographic dye flow, we also generated digital subtraction angiograms by subtracting out an unenhanced key frame image from successive images (Fig. 1, Movies 2 and 4) (Choma et al., 2011). Following injection, dye was transported into the pre-pupal heart from the extracellular-extravascular space through paired ostia inflow tracts. At this developmental stage, the *Drosophila* heart has three pairs of ostia, one pair for each abdominal segment (A5, A6 and A7) of the heart (Fig. 1A). Two aspects of the transport process from the extracellular-extravascular space into the heart were consistent with low Reynolds number creeping flow: first, flow was in lockstep with the cardiac cycle, suggesting a lack of significant inertial-driven flow; second, dye inflow through an individual ostium did not fill the entire heart but rather remained local to the inflow segment. This segmental-type filling occurs in the absence of formal intracardiac valves and is attributable to low Reynolds number physiology. This is important from a flow velocimetry perspective since it enables the formation of distinct interfaces between dye and hemolymph (the circulatory fluid in *D. melanogaster*), which can be tracked over time within the heart and aorta. As the heart wall began to contract, the dye-hemolymph interface visibly deformed into a parabolic profile (Movie 5). Tracking the interface revealed that intra-cardiac and intra-aortic flow velocities are in the 10 mm/s regime. Imaging at a few to several hundred frames per second is necessary to visualize and quantify flows that are in this speed regime and justifies the use of frame rates well above video rate.

### Optical angiography and OCT reveal pathological micro- and mesoscopic hypoplastic aortic physiology in *hdp^2^* (troponin I) mutants

**Fig. 2.**
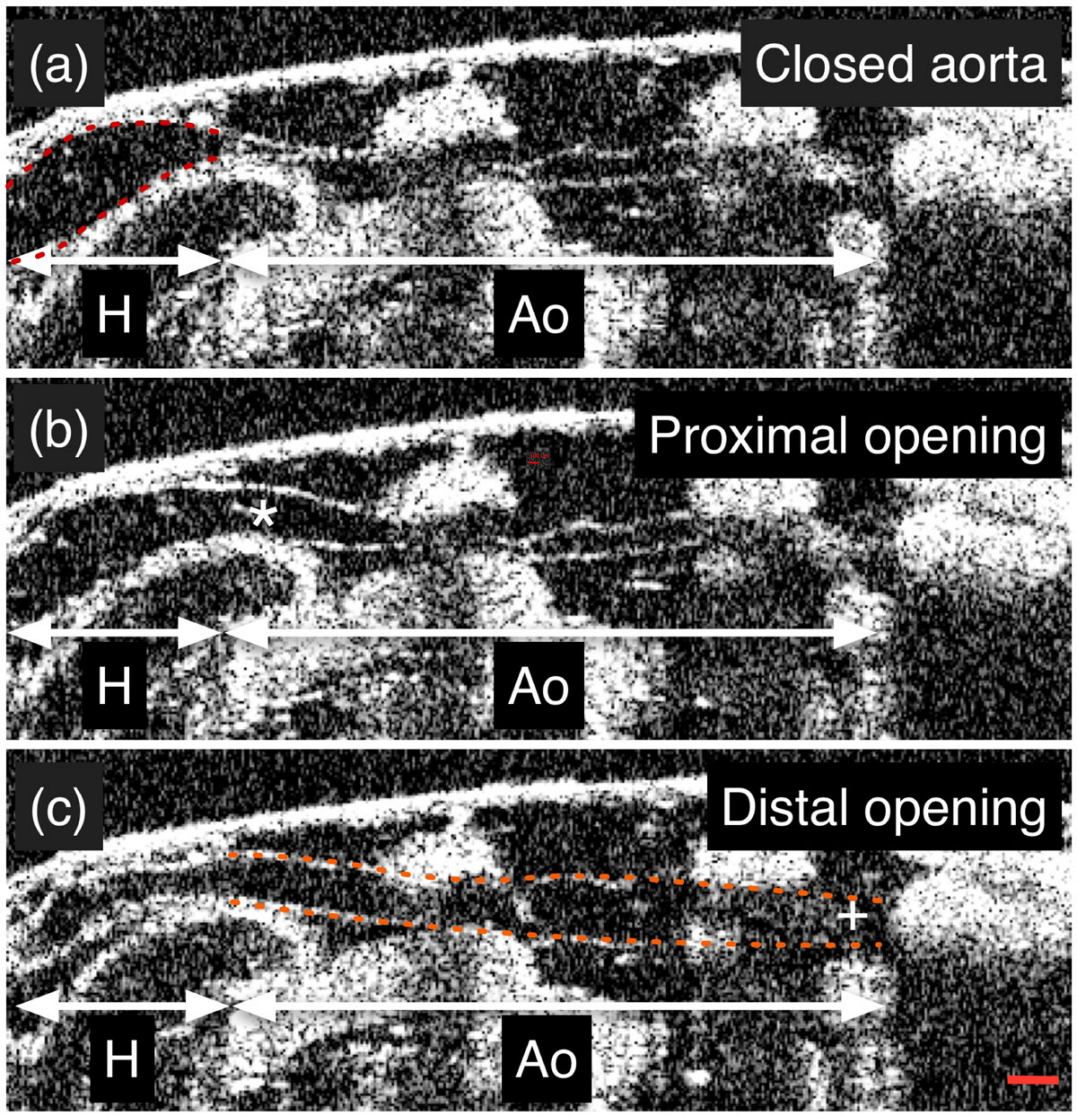
**OCT-based assessment of aortic pulse wave velocity.** (A) The aorta is closed immediately prior to the initiation of cardiac systole. (B) The proximal (*) aorta opens shortly after the initiation of cardiac systole. (C) The distal (+) aorta opens some time (Δt) later. Pulse wave velocity is given by PWV=Δx/Δt, where Δx is the distance between the proximal and distal aorta, and Δt is the time it takes for the pulse wave to travel Δx. H, heart; Ao, aorta. See also Movie 6. Red dashed line, cardiac wall; orange dashed line, aortic wall. Scale bar: 100 um.

We next investigated aortic flow and wall dynamics during aortic filling and opening in response to cardiac systole in *hdp^2^*, troponin I-mutant *Drosophila.* Assessment of hemolymph flow velocity by OCT Doppler is technically difficult because the plane of flow is orthogonal to the imaging plane, and because there are hemocytes in the hemolymph. Therefore, our initial investigations in the present study focused on estimating aortic flow velocity using high-speed dye angiography in both *hdp^2^* and *OreR* pre-pupae. After confirming the presence of a mutant heart wall motion defect (Table 1), peak aortic flow velocity was quantified using high-speed dye angiography and was determined to be higher in *hdp^2^* compared to *OreR* aortas (*hdp^2^*: 19 mm/s±3.8 mm/s, *n*=10; *OreR*: 12 mm/s±3.7 mm/s, *n*=8; *P*<0.005 using Wilcoxon rank-sum test). However, differentially altered fluid dynamics due to dye injection could not be excluded. Thus, we used a complementary OCT-based method to validate the findings. Specifically, we employed dynamic structural OCT imaging to perform aortic pulse wave velocity (PWV) measurements to provide an index of *in vivo* aortic stiffness, in *hdp^2^* and two, independent control strains, *OreR*, and *Canton S* (cs) (Fig. 2). OCT movies taken along the long axis (anterior–posterior axis) were used to estimate the rate of pressure transmission in the aorta. PWV was estimated by taking the quotient of the distance between the proximal and distal aorta and the time difference between the initiation of proximal and distal aortic opening. *hdp^2^* aortas had faster PWVs compared to both *OreR* and cs controls, consistent with increased aortic flow velocity in mutant *hdp^2^* compared to *OreR* as quantified using dye angiography (Table 2).

**Table 1.**
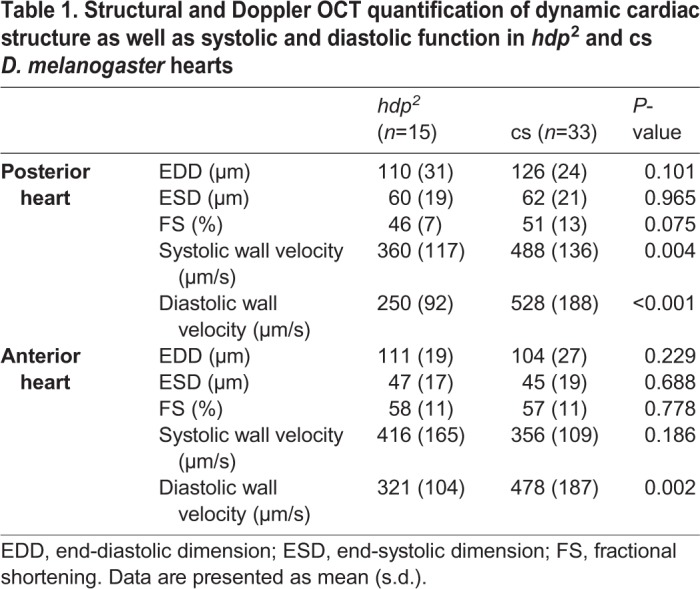
**Structural and Doppler OCT quantification of dynamic cardiac structure as well as systolic and diastolic function in *hdp*^2^ and cs *D. melanogaster* hearts**

**Table 2.**
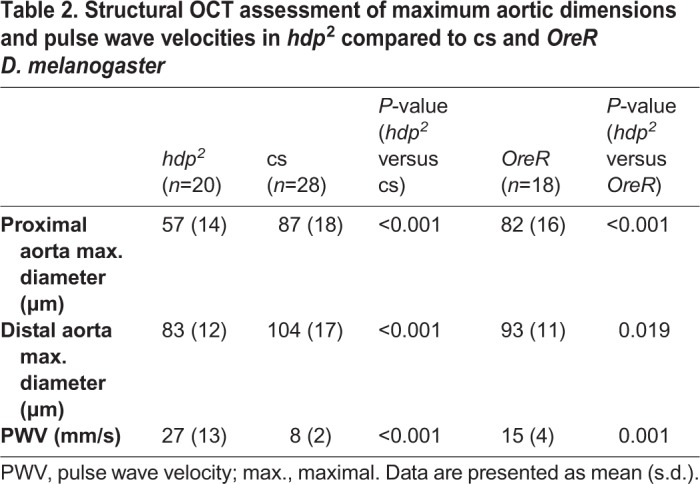
**Structural OCT assessment of maximum aortic dimensions and pulse wave velocities in *hdp*^2^ compared to cs and *OreRD. melanogaster***

With evidence that *hdp^2^* mutants have increased aortic flow velocity in the setting of preserved cardiac outflow tract (anterior heart) dimensions and fractional shortening, we hypothesized that *hdp^2^* aortas have defective opening and filling in response to flow generated by cardiac systole. The possibility that the troponin I mutation causes impaired aortic opening and filling is supported by the fact that the *D. melanogaster* heart and aorta are composed of striated myocytes, and thus sarcomeric defects may alter cardiomyocyte and aortic physiology. OCT was used to quantify peak aortic diameters after cardiac systole, and we found that *hdp^2^* aortas were hypoplastic (Table 2). Thus, multimodal imaging at micro- and mesoscopic size scales yielded physiological data consistent with aortic hypoplasia in *hdp^2^* pre-pupae.

### Molecular-specific imaging using confocal microscopy reveals nano/microscopic as well as mesoscopic defects in *hdp^2^* aortic myofibrils

**Fig. 3.**
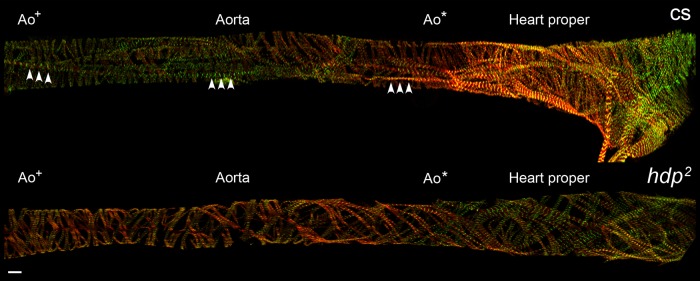
**Confocal imaging of myofibrils in *D. melanogaster* larval hearts and aortas.** Wild-type and mutant *Tung-GFP*-expressing (green) third instar larvae hearts were exposed, relaxed, fixed and stained with Alexa 594-phalloidin (red) and imaged at 40× magnification. Relative to *hdp^2^*, cs cardiomyocytes and aortic cells overall contained more contractile material and were characterized by an abundance of myofibrils running parallel to the longitudinal axis of the heart proper and aorta (arrowheads). Such longitudinally-oriented myofibrils, extending the length of *hdp^2^* aortas, were infrequently resolved. Ao*, proximal aorta; Ao^+^, distal aorta. Scale bar: 10 µm.

**Fig. 4.**
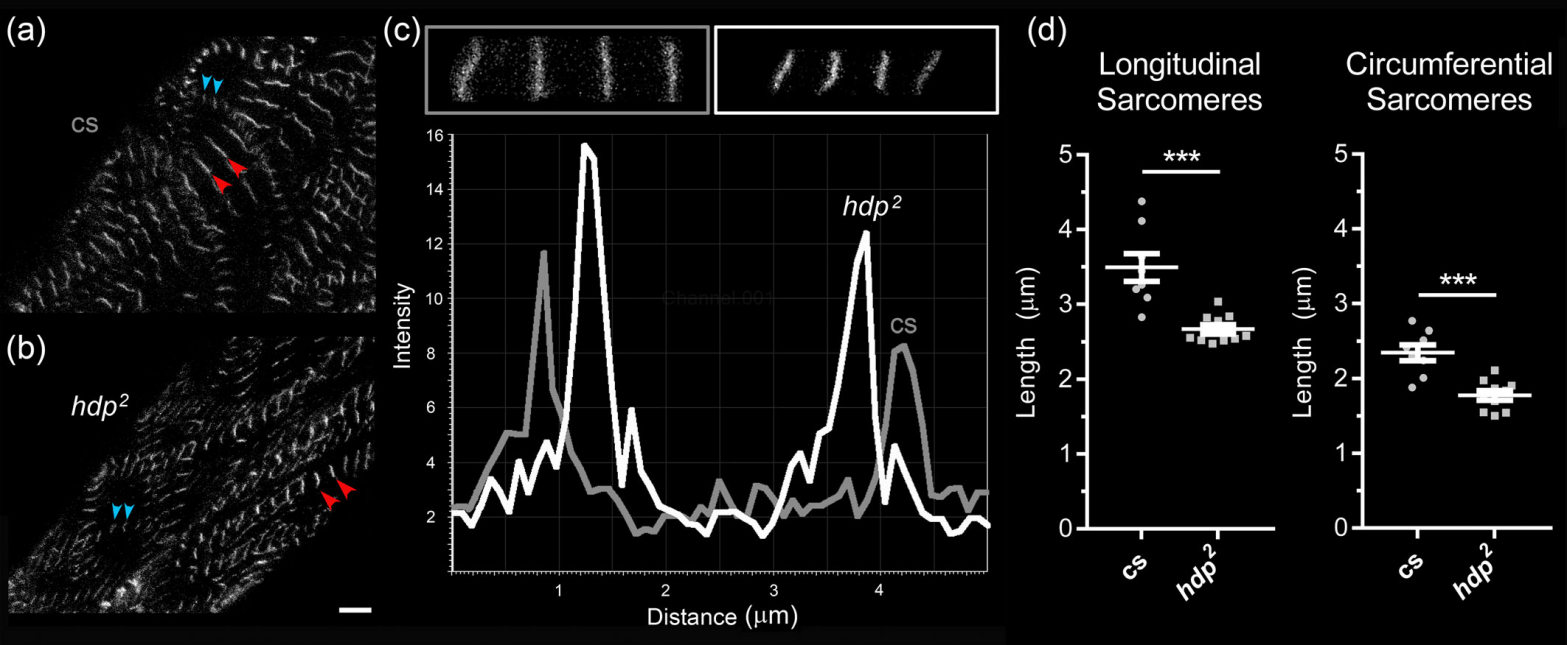
**Quantification of sarcomere length in cs and *hdp^2^* pre-pupae aortas.** (A,B) Myocytes from *Tung-GFP*-expressing cs and *hdp^2^* proximal aortas. Red and blue arrowheads delineate individual sarcomeres from longitudinally and circumferentially-oriented myofibrils, respectively. Scale bar: 10 µm. (C) Representative images of consecutive sarcomeres (top) and plots of fluorescence intensity as a function of distance along individual, longitudinally-arranged myofibrils (bottom) from cs (grey) and *hdp^2^* (white) proximal aortas. Sarcomere length was determined by measuring the distance between successive peaks in intensity. (D) Both longitudinal and circumferential sarcomere lengths are shorter in *hdp^2^* pre-pupae aortas. ***P<0.001 using Wilcoxon Rank-Sum test.

To correlate the functional abnormalities in *hdp^2^* aortas with microstructural changes, we performed confocal microscopy of Alexa 594-phalloidin-labeled aortas of *hdp^2^* and cs third instar larvae expressing *Tung-GFP*, which encodes a sarcomeric Z-disc-associated protein tagged with GFP (Fig. 3). A blinded observer (C.G.W.) was able to distinguish *hdp^2^* mutant aortas from cs in 23 of 24 images. The main distinguishing feature was that the *hdp^2^* vessels contained fewer or even lacked longitudinally-oriented myofibrils compared to wild-type aortas (Fig. 3). Additionally, sarcomere length was significantly shorter in *hdp^2^* compared to cs for circumferential (1.74±0.18 µm versus 2.35±0.3 µm, *P*<0.001 using Wilcoxon Rank-Sum test) and longitudinal (2.67±0.19 µm versus 3.49±0.53 µm, *P*<0.001 using Wilcoxon Rank-Sum test) myofibrils using individual mean sarcomeric length of 8–10 flies per genotype (Fig. 4). Circumferential sarcomeres were shorter than longitudinal sarcomeres in both genotypes (*P*<0.001 using Wilcoxon Signed-Rank Test).

In summary, the *hdp^2^* mutation is associated with aortic and heart tube functional perturbations in pre-pupae as well as nano- to microscopic structural abnormalities as evidenced by shorter sarcomere length and paucity of longitudinal myofibrils in third instar larvae.

## DISCUSSION

We demonstrate a novel approach to comprehensively, quantitatively and dynamically assess cardiovascular pathology and pathophysiology using multiscalar, multimodal imaging methodologies. We show for the first time that aortic pathology and heart tube dysfunction coexist and likely interact in *hdp^2^* pre-pupae, which express a myopathy-inducing troponin I variant. Shortened, hypercontractile sarcomeres in the mutants plausibly lead to altered dynamic heart function as well as a hypoplastic aortic phenotype. Furthermore, aortic hypoplasia leads to increased hemolymph flow velocity and increased afterload, which may additionally affect cardiac function similar to humans. Integration of multiple anatomic and functional cardiovascular parameters using multimodal multiscalar optical imaging techniques leads to a unified pathophysiological picture from the molecular through the organ level. This model may become valuable in the search of pharmacological treatment strategies for human cardiomyopathy and modification of vascular–ventricular interaction.

Our new understanding of integrative sarcomeric and cardiovascular *D. melanogaster* physiology has implications for studying and understanding vertebrate embryonic heart physiology. In particular, the early embryonic ventricle pumps into a myocardial chamber-like vessel, resembling the outflow tract in chick embryos (Movie 7; Deniz et al., 2012). This raises the possibility that altered myocardial properties alter outflow tract impedance and, therefore, ventricular afterload. Changes in afterload may affect intracardiac hemodynamic force, an important epigenetic factor in heart development. Thus, in addition to revealing novel pathophysiology, the pairing of sophisticated optical imaging with genetically altered *D. melanogaster* suggests a novel path for future investigation in the study of vertebrate heart development and congenital heart defects.

Additionally, our results provide insight into cardiovascular cytoarchitecture in both health and disease. Wild-type pre-pupal hearts and aortas were characterized by several contiguous, longitudinally-oriented myofibrils that apparently ‘overlapped’ with circumferentially-oriented myofibrils – to our knowledge this has not been described in *D. melanogaster*. Of note though, a histologic assessment of the larval heart tube has revealed this myoarchitecture previously (Monier et al., 2005). Since myofibrils typically extend from the bipolar ends, down the long-axis of myocytes, the observation of perpendicularly-oriented structures within the same plane suggests the presence of distinct, overlapping muscle cells. Each cell likely contains myofibrils running in a uniform direction dictated by the orientation of the myocyte. The coexistence of potentially distinct muscle layers is also supported by longer sarcomere lengths along longitudinal relative to circumferential myofibrils, consistent with a discrete myofibrillar architecture to meet the unique contractile demands of potentially anisotropically-layered cell types. In contrast, the *hdp^2^* pre-pupae aorta had a qualitatively and quantitatively distinct myofibrillar architecture. There was a paucity of longitudinally-oriented myofibrils, and both longitudinally- and perpendicularly-oriented aortic myofibrils exhibited significantly shorter sarcomere lengths when maintained under low-calcium conditions. These results are consistent with earlier studies, which demonstrated that, despite a lack of single molecule data, the *hdp^2^* mutation induced a myosin-dependent, hypercontractile state and enhanced calcium-sensitivity of contractile activation due to an impaired ability of troponin I to properly promote tropomyosin-dependent relaxation (Beall and Fyrberg, 1991; Cammarato et al., 2004; Vikhorev et al., 2010). Similarly, resting sarcomeric length was found to be decreased in isolated myocytes expressing troponin I harboring a human restrictive cardiomyopathy-causing mutation (Davis et al., 2012). The mutant troponin I-dependent mechanical tone, caused by acute remodeling to a quasi-contracted myocyte state, likely contributes to elevated myocardial stiffness observed in these patients and functionally analogous mutations expressed during development may perturb outflow tract physiology and cardiogenesis. Thus, our data suggest that in the *hdp^2^ D. melanogaster* pre-pupal aorta, which is composed of striated muscle, excessive dysinhibited acto-myosin cycling results in shortened sarcomeres and elevated basal tone, which likely contributes to the hypoplastic aortic phenotype as well as to increased flow velocities*.*

Mutations in the human *wupA* ortholog *TNNI3* cause hypertrophic, dilated or restrictive cardiomyopathy, yet a human mutation-specific genotype-phenotype correlation, as identified here for *hdp^2^*, has not been established for *TNNI3* mutations (Mogensen et al., 2015). Physiologically, the *hdp^2^* cardiovascular phenotype has several additional parallels to human hypertrophic and restrictive cardiomyopathy (*TNNI3* OMIM #191044), which are characterized by diastolic dysfunction, subtle systolic abnormalities and sarcomeric contractile dysfunction in the presence of generally preserved systolic function, increased outflow tract velocities due to outflow tract obstruction, and increased aortic stiffness (Austin et al., 2010; Nagueh et al., 2003; van Dijk et al., 2012). It has been suggested that vascular–ventricular interaction may contribute to cardiac dysfunction in patients with hypertrophic cardiomyopathy (HCM) (Austin et al., 2010). Vascular–ventricular interaction describes the dependence of cardiac function on the arterial system into which the heart ejects. With increased aortic stiffness, the PWV is increased, and the reflected pulse wave returns to the heart early, increasing late systolic afterload, which affects thick–thin myofilament interactions and leads to diastolic and ultimately systolic dysfunction (Borlaug and Kass, 2011; Lombardi et al., 2013). In our *D. melanogaster* model, the aorta is hypoplastic, and flow as well as PWVs are increased, consistent with obstructive physiology. Similar timing of arterial pressure and flow curves has been demonstrated for the frog previously (Langille and Jones, 1977), distinguishing the primitive from the more complex mammalian cardiovascular physiology. The increased afterload in *hdp^2^* may contribute to diastolic dysfunction and mild systolic dysfunction. While we had previously demonstrated cardiac dysfunction in *hdp^2^* compared to *OreR* as controls (Choma et al., 2011), we herein validated those results by using cs as an additional control. Similarly, diastolic dysfunction has been described in mutant *upheld^101^*, the *D. melanogaster* ortholog to human cardiac Troponin T (Viswanathan et al., 2014). We suggest that the *hdp^2^* pre-pupal heart is exposed to increased afterload due to aortic hypoplasia, and that impaired vascular-cardiac tube interaction may in part be responsible for diastolic and subtle systolic dysfunction.

A limitation of the current study is the lack of the distinction if aortic and myocardial structural and functional abnormalities are solely dependent on the *hdp^2^* genotype or if vascular–heart tube interaction may contribute to cardiac dysfunction in mutant pre-pupae. Further, *D. melanogaster* has a tubular heart and open cardiovascular system with the aorta containing striated muscle. This may not perfectly mirror complex human cardiovascular physiology, however, due to its low cost and short generation times, *D. melanogaster* is an attractive model to study genetic and possibly pharmacological modification of cardiovascular physiology in the future (Dar et al., 2012).

In conclusion, this study demonstrates the utility of *D. melanogaster* to investigate vascular and cardiac chamber function. We identified physiological and structural changes in *hdp^2^* mutant hearts and aortas using multiple optical imaging modalities and contrast mechanisms. Overall, perturbations in the cytoarchitecture and shorter sarcomeres in *hdp^2^* mutant larvae lead to smaller, stiffer aortas, which result in increased flow velocity determined by OCT and angiography. *hdp^2^* mutant hearts also displayed diastolic and latent systolic dysfunction. It remains to be seen how far vascular–myocardial interactions contribute to the development of diastolic and ultimately systolic dysfunction in *D. melanogaster* and in patients with HCM. In the future, our comprehensive imaging approach may be used to study therapeutic targets for human cardiac disease (Dar et al., 2012).

## MATERIALS AND METHODS

### *Drosophila melanogaster* culture and pre-pupal selection

Wild-type cs and *OreR*, mutant *hdp^2^*, and *Tung189* (*Tung-GFP*) (Morin et al., 2001) *D. melanogaster* were raised on reconstituted dry medium (Carolina Biological Supply, Burlington, USA). The genetic background of *hdp^2^* mutant flies is cs. All flies were cultured and imaged at room temperature. Pre-pupae were identified and prepared for imaging as previously described (Choma et al., 2011).

For larval heart tube and aorta imaging experiments, males expressing *Tung-GFP* were crossed with virgin *hdp^2^* mutant flies or with cs virgins as controls. Because the *hdp^2^* mutation is located on the X-chromosome, male progeny from both crosses were selected for imaging to ensure *hdp^2^* larvae were hemizygous for the troponin I mutation and expressed *Tung-GFP*.

### Structural and Doppler optical coherence tomography (OCT) imaging

Quantitative comparisons of *OreR* and *hdp^2^* hearts were made using a commercially available OCT system (Thorlabs, Newton, USA) with a 1325 nm center wavelength. Post-processing and chamber measurements were performed using MATLAB (Mathworks, Natick, USA) and ImageJ (Wayne Rasband, National Institutes of Health).

End systolic and diastolic diameters for an individual specimen were reported as the average diameters measured for three distinct heartbeats. Fractional shortening was calculated as (EDD–ESD)/EDD. Here, EDD is the end-diastolic diameter and ESD is the end-systolic diameter. For Doppler imaging, conversion from measured Doppler shift (f_d_) to velocity (v) used the standard Doppler equation for echo-based Doppler velocimetry (f_d_=2vcos(θ)nν/c; θ, Doppler angle; n, tissue optical index; ν, optical frequency; c, free-space vacuum speed of light). This measurement was performed for peak systolic and diastolic tissue velocities of the ventral heart wall for each M-mode recording, and averaged over three beats. The heart axis was positioned so that wall motion was parallel to the optical axis (i.e. Doppler angle of 0°).

### Aortic imaging

The heart and aorta were imaged along the long axis (anterior–posterior axis). PWV was defined as the ratio of the distance between the most proximal and distal visible location of the aorta, and the time taken for the pulse wave to travel this distance. Short axis (left–right) images of the proximal and distal aorta were obtained. Maximal systolic dimensions were determined and averaged over three beats.

For angiography, a solution of Brilliant Blue G and normal saline was injected into the extracellular–extravascular space (EES) using a custom-pulled glass micropipette attached to a pressurized injection system. Dye angiography was recorded using a high-speed camera at 250 or 500 frames per second (Redlake/IDT Y4 Lite). Digital subtraction angiography of intracardiac, aortic and EES microfluidic flow were performed as previously described (Choma et al., 2011).

Male cs×*Tung-GFP* and *hdp^2^*×*Tung-GFP* larval hearts were dissected as described with minor modifications (Cooper et al., 2009). Briefly, larvae were placed in a drop of cold, artificial adult *D. melanogaster* hemolymph, rotated onto their ventral side, and pins inserted at both ends of each larva. An incision was made from one end to the other, being careful not to touch the heart tube located on the dorsal side of the preparation. Four pins were placed at each corner of the cuticle to expose the heart tube and aorta. Contractions were inhibited with EGTA. Before removing the internal organs, larvae were fixed for 30 min in 4% paraformaldehyde. Preparations were washed three times in 1× PBS with 0.1% Triton X-100 and once in 1× PBS. Organs were then removed to expose the larval heart tube and aorta. Preparations were incubated at 4°C in 1:250 Alexa Fluor 594 phalloidin (Life Technologies) for 24 h, washed three times in 1× PBST and once in 1× PBS. Preparations were then mounted as described (Alayari et al., 2009).

Larval hearts were visualized and imaged on a Leica TCS SPE RGBV confocal microscope fitted with 40× and 63× oil immersion lenses. Individual images at adjacent locations over each heart and aorta were spliced together using Adobe Photoshop (version 9.0). Five to 30 well-resolved sarcomeres were measured per animal using LAS EZ software and averaged. The average longitudinal and circumferential sarcomere lengths from 8 to 10 animals were then averaged per genotype and compared.

### Statistics

Continuous variables are expressed as mean (standard deviation). Wilcoxon Rank-Sum test and Wilcoxon Signed-Rank test were used as appropriate to compare continuous variables between groups.

## Supplementary Material

Supplementary information
