# Supplementary material for: Multi-modal and multiscale imaging approaches reveal novel cardiovascular pathophysiology in Drosophila melanogaster
Source: Biol Open. 2019 Aug 15;8(8):bio044339. doi: 10.1242/bio.044339 (PMC6737974; doi:10.1242/bio.044339)
Supplement: Supplementary information [file biolopen-8-044339-s1.pdf]

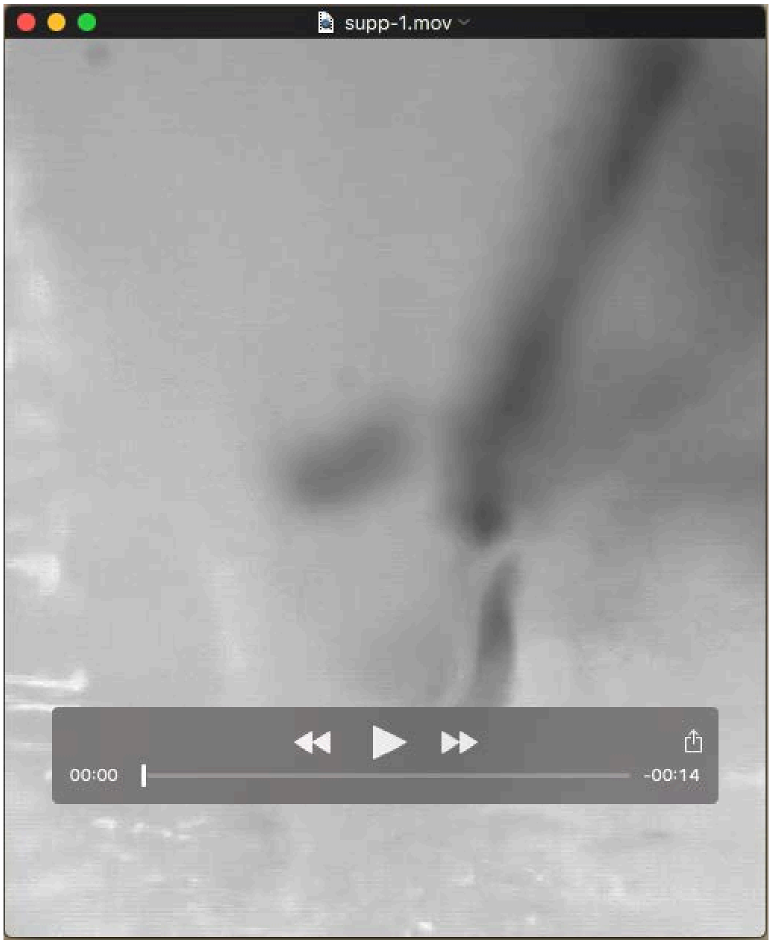

Movie 1

High-speed optical angiography of pre-pupal *D. melanogaster* cardiac fluid flow. Unilateral dye inflow though A5 ostia (5th abdominal segment; see also Fig. 1b). The videomicroscopic movie was acquired at 500 frames per second (fps) and is displayed at 25 fps (20x slo-mo).

Biology Open • Supplementary information

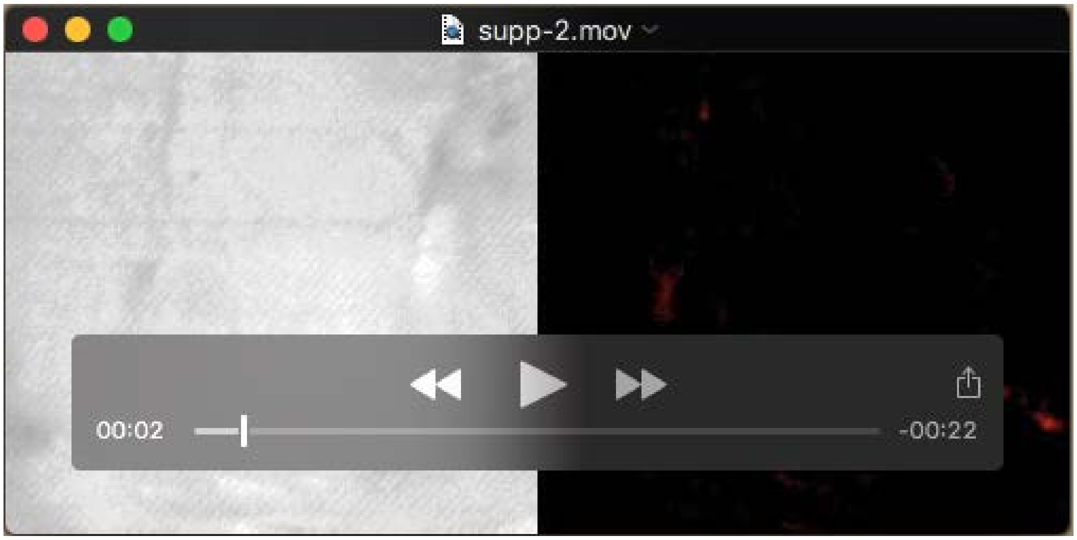

Movie 2

High-speed optical angiography of pre-pupal *D. melanogaster* cardiac fluid flow. Unilateral dye inflow though A6 ostia (6th abdominal segment; see also Fig. 1c). The videomicroscopic movie was acquired at 250 fps and is displayed at 12.5 fps (20x slo-mo).

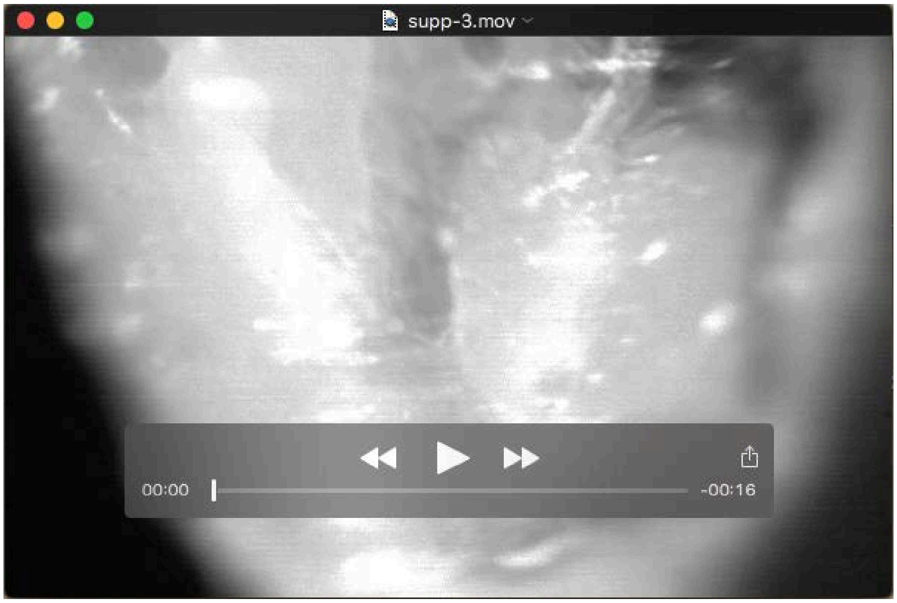

Movie 3

High-speed optical angiography of pre-pupal *D. melanogaster* cardiac fluid flow. Unilateral dye inflow though A7 ostia (7th abdominal segment; see also Fig. 1d). Residual The videomicroscopic movie was acquired at 500 frames per second (fps) and is displayed at 25 fps (20x slo-mo).

Biology Open • Supplementary information

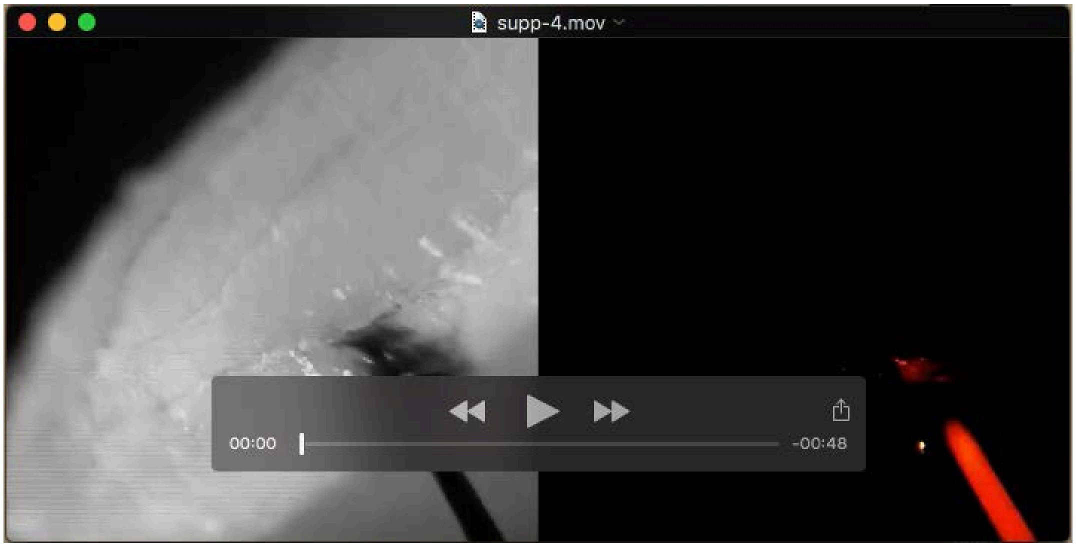

Movie 4

High-speed optical angiography (left panel) and digital subtraction angiography (right panel) of pre-pupal *D. melanogaster* cardiac and aortic fluid flow. See also Fig. 1e-l. The videomicroscopic movie was acquired at 250 fps and is displayed at 12.5 fps (20x slo-mo).

Biology Open • Supplementary information

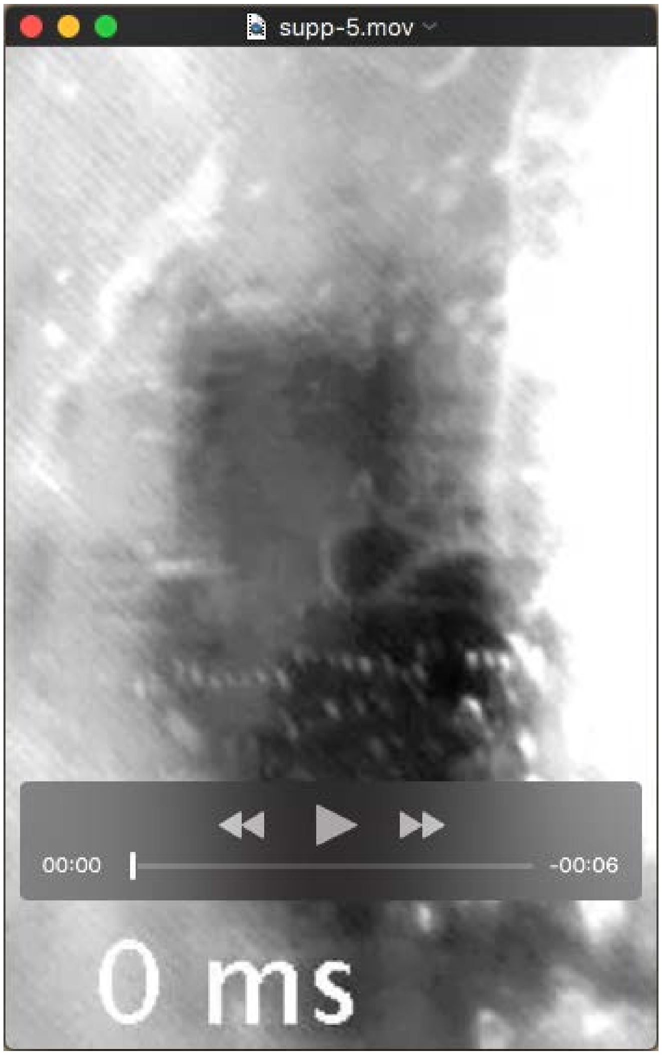

Movie 5

Shear force-mediated deformation of intracardiac dye/hemolymph interfaces visualized using dye angiography. The development of parabolic flow can be seen over the course of the video. The videomicroscopic movie was acquired at 500 frames per second (fps) and is displayed at 2 fps (250x slo-mo).

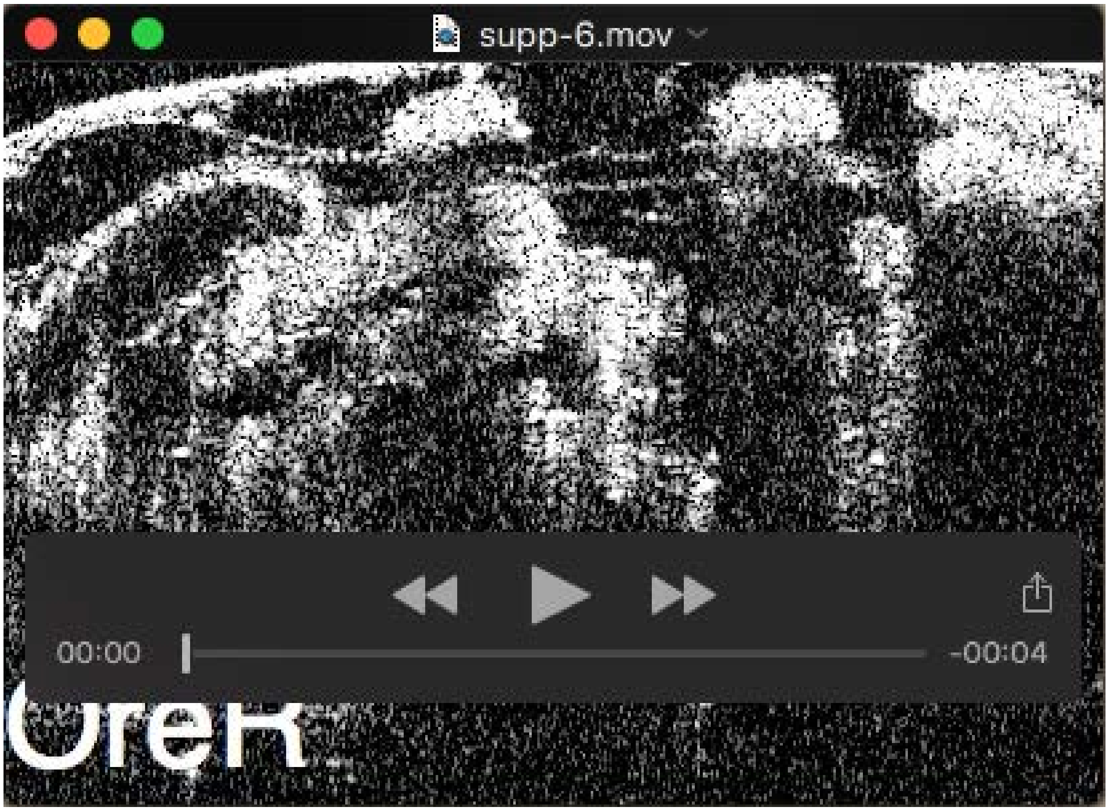

Movie 6

OCT-based assessment of aortic pulse wave velocity using OreR as an example. The aorta is closed immediately prior to the initiation of cardiac systole. The proximal aorta opens shortly after the initiation of cardiac systole, and opens more distally as the pulse wave propagates. Pulse wave velocity was defined as the ratio between the distance between the beginning of proximal and distal aortic opening, and the time between aortic opening at both points. Acquired at 125 fps, displayed at 15 fps (8.3x slo-mo).

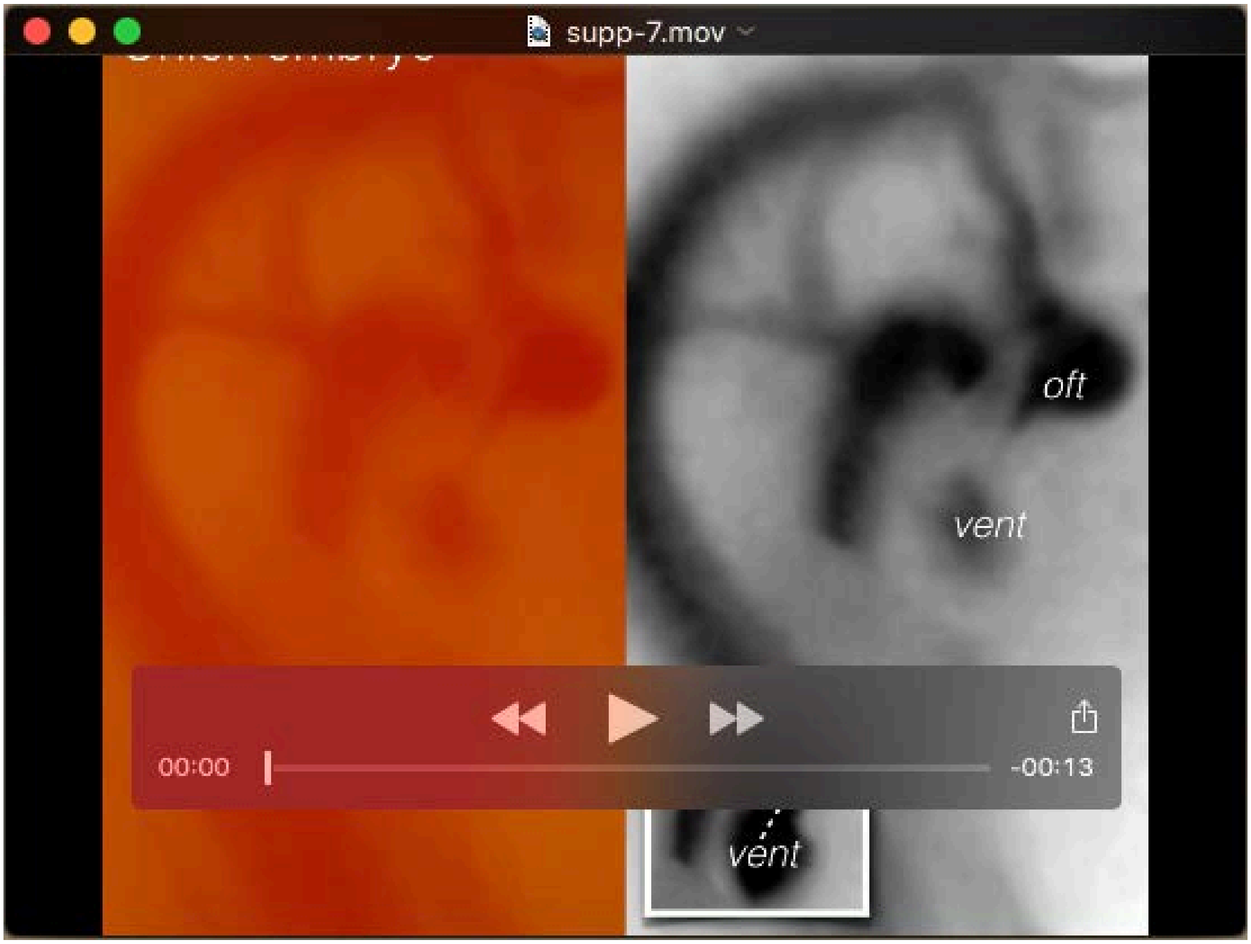

Movie 7

Videomicroscopy (left panel) and hemoglobin contrast subtraction angiography (HCSA; right panel)(Deniz et al. 2012) movies of blood flow in a Hamburger-Hamilton stage 18 chick embryo. oft, outflow tract; vent, ventricle. Movie was acquired at 30 fps and played back at 6 fps (5x slo-mo).

Biology Open • Supplementary information
